# Uncovering heterogeneity in South African students’ mathematics competence: latent profile analysis and multinomial logistic regression

**DOI:** 10.3389/fpsyg.2026.1850152

**Published:** 2026-09-17

**Authors:** Millicent Aziku, Dahman Tahri, Denis Djekourmane

**Affiliations:** 1 Shaanxi Normal University, Xi’an, China; 2 Faculty of Education, Beijing Normal University, Beijing, China

**Keywords:** large-scale assessment, latent profile analysis, mathematics competence, South Africa, TIMSS

## Abstract

**Introduction:**

Mathematics competence is multidimensional, and students may demonstrate different patterns of performance across mathematical content and cognitive domains. This study examined heterogeneity in mathematics competence among South African Grade 8 students and investigated factors associated with membership in distinct mathematics competence profiles. Guided by Bronfenbrenner’s ecological theory of human development, the study considered individual characteristics and features of students’ immediate home and school environments.

**Methods:**

The study used data from 11,012 South African Grade 8 students who participated in the Trends in International Mathematics and Science Study (TIMSS) 2023. Latent Profile Analysis (LPA) was used to identify distinct mathematics competence profiles across seven domains: Number, Algebra, Geometry, Data and Probability, Knowing, Applying, and Reasoning. A three-step multinomial logistic regression was subsequently conducted to examine individual- and contextual-level factors associated with profile membership.

**Results:**

Four mathematics competence profiles were identified: Low, Lower-middle, Upper-middle, and High competence. Profile membership was differentially associated with students’ characteristics and immediate learning environments. In particular, mathematics confidence, home educational resources, school belonging, and bullying were notably associated with membership in the higher-competence profiles, although the magnitude and direction of these associations varied across profiles.

**Discussion:**

The findings demonstrate substantial heterogeneity in students’ mathematics competence and suggest that mathematics performance is associated with a combination of individual and contextual factors. The results highlight the importance of recognizing differentiated patterns of mathematics competence when designing educational policies and support strategies and of considering students’ home and school environments alongside individual characteristics.

## 1 Introduction

Owing to the swift global economic transformation, which saw many sectors demanding STEM (science, technology, engineering, and mathematics) experts, developing mathematics competence has become a prerequisite to keeping abreast of modern society. Mathematics is widely regarded as a crucial factor in accessing science-related careers, prompting countries to implement measures aimed at improving students’ mathematical competencies (Bansilal and Lephoto, 2022). South Africa, just like other Sub-Saharan African countries, has been battling with revamping young students’ mathematics competence to prepare them for potential jobs (Kattamaney, 2025). Despite the recognition of the central role of mathematics competence, evidence from large-scale assessments, such as the Trends in International Mathematics and Science Study (TIMSS), demonstrates that the performance of South African students in mathematics assessments is consistently lower compared to many other countries. In TIMSS 2023, South African Grade 8 students’ overall mathematics performance was among the last five countries (Von Davier et al., 2024). This persistent gap has attracted concerns about the possible factors that impact students’ mathematical competence in South Africa.

Existing research suggests that mathematical competence is shaped by multiple factors at the person- and context-levels. For instance, scholarship suggests that holding all factors constant, girls possess lower mathematics competence compared to their male counterparts (Aldrup et al., 2020; Avci et al., 2025; Gutfleisch and Kogan, 2024; Liu et al., 2026). However, this assertion does not apply to the general group of South African students. In fact, girls reportedly performed higher on mathematics tasks compared to boys (Abin et al., 2020). Meanwhile, studies proposed building students’ positive attitudes toward mathematics, such as confidence, interest, enjoyment, etc. (Grabowska et al., 2025; Nakamoto et al., 2025; Rican et al., 2022). Yet, most previous research has treated students as a homogeneous group, overlooking the possibility that predictors of mathematics competence may operate differently across students with varying levels of competence. This limitation calls for a more nuanced approach that recognizes the heterogeneity in students’ competence profiles.

To bridge this literature gap, the present study uses Latent Profile Analysis (LPA) to classify students according to their mathematics competence and examines how person- and context-level characteristics are associated with mathematics-competence profile membership, guided by Bronfenbrenner’s (1979) ecological theory of human development. By doing so, the study aims to provide a deeper understanding of the complex interplay of variables that shape mathematical competence among South African students. Such understanding is crucial for designing targeted policy interventions and equitable educational policies that address the specific needs of students at different competency levels. The study is guided by the following research questions:

1 What distinct profiles of mathematics competence can be identified among South African Grade 8 students?
2 To what extent are person- and context-level characteristics associated with membership in the identified profiles?

## 2 Literature review

### 2.1 South African students’ performance in TIMSS 2023

TIMSS is a large-scale international assessment that is conducted once every 4 years by the International Association for the Evaluation of Educational Achievement (IEA) to evaluate the performance of Grade 4 and 8 students in science and mathematics (Fishbein et al., 2025). Apart from assessing the students’ performance, TIMSS also collects data on certain factors that could influence the achievement of students, such as instruction, school environment, and home resources, among others. The newest cycle of TIMSS is the 2023 wave. South Africa’s Grade 8 students’ overall performance in TIMSS 2023 was relatively low. Out of 46 countries that participated in the assessment, South African students’ mathematics performance was in the last five, with an overall mean of 397 (Von Davier et al., 2024). This result calls for a more profound investigation into possible variables that influence the students’ competence.

### 2.2 Theoretical framework

The current study is grounded in Bronfenbrenner’s (1979) ecological theory of human development, which conceptualizes development as emerging from the dynamic interaction between individuals and the environments in which they live and learn. Guided by this perspective, the study considers mathematics competence in relation to both person-level characteristics and immediate contextual conditions. Person-level characteristics include sex, age, and students’ mathematics-related attitudes, namely confidence in mathematics, enjoyment of mathematics, and the value they place on it. The contextual component focuses on students’ immediate learning environments, where home educational resources represent the home context, and the school context includes factors such as instructional clarity, disorderly behavior, school belonging, and bullying. The ecological framework is used to guide interpretation of the factors associated with profile membership, examining whether person-level characteristics and features of students’ immediate home and school environments differentiate membership across the identified competence profiles after LPA. This approach positions mathematics competence within the broader ecological environments in which students learn while allowing the profiles themselves to be determined empirically.

#### 2.2.1 Person-level variables and mathematics competence

Globally, students’ mathematics competence has been measured in relation to personal characteristics such as sex, age, and attitudes toward mathematics (confidence, value, and enjoyment). For example, a study by Bansilal and Lephoto (2022), which investigated how certain student-level variables affected South African students’ mathematical competence using TIMSS 2015, demonstrated that when learners are older than the required age for their grade, their mathematical competence reduces. By contrast, another study reported that student age had a positive association with their reasoning capabilities, thereby improving their competency level (Almomani et al., 2018). This finding is reinforced by a recent study, which referred to age as a crucial variable for mathematics improvement (Akin, 2025). Apart from students’ age, the role of their sex has been critically examined by previous scholars. While some studies indicated that boys displayed higher mathematics competence than girls (Arroyo-Barriguete et al., 2023; Chen, 2022), a study that employed Blinder-Oaxaca decomposition to evaluate the sex difference in mathematics competence recorded higher competence in favor of South African girls (Hofmeyr, 2022). Sibanda and Naidoo (2023) reported favorable mathematics competence for girls compared to boys while explaining that boys only performed better with regard to questions that required reasoning than knowing and applying. Still, Wilson Fadiji and Reddy (2023) found no significant relationship between South African girls and boys regarding their mathematics competence.

Similarly, substantial evidence establishes positive relationships between positive attitudes toward mathematics and mathematical competence. For instance, a study by Maree et al. (2013), which evaluated how study behavior influenced students’ mathematics competence, found a significant positive correlation in that students who developed positive behavior toward mathematics learning scored higher on mathematics tasks. Specifically, the inner enjoyment that students derive from performing mathematical tasks triggers their urge to explore more, hence improving their competence (Moyano et al., 2020). The enjoyment is a result of the emotions that students attach to learning mathematics, which consequently predicts their mathematics performance (St Omer et al., 2023). Relatedly, previous scholarship consistently highlighted a positive relationship between mathematics confidence and competence (Aziku et al., 2026; Flanagan and Einarson, 2017; Nakamoto et al., 2025). However, a study revealed that for girls, their mathematics confidence positively correlated with their scores, while boys overestimated their confidence, leading to adverse correlation (Raabe and Block, 2024). A study found that, on average, mathematics-related attitudes explained about 20% of the variance in mathematics competence among Grade 4 and Grade 8 students who participated in the TIMSS 2019 assessment (Yagan, 2021).

The conflicting results demonstrated in existing studies regarding the relationships between these person-level variables and mathematics competence could stem from the treatment of students as a homogeneous sample without exploring the heterogeneity among them. Variations in students’ mathematical competence may mean that the same factors have different associations (Zeng, 2024). Additionally, although the positive relationship between mathematics attitude and competence is well-established, it is not well-known whether the direction and magnitude are uniform for students at all levels of competence. This current study suggests that learners should not be simply regarded as a homogeneous sample. The study therefore hypothesized that for students with varied patterns of mathematical competence, the relationship between their personal characteristics and mathematical competence may differ.

#### 2.2.2 Context-level variables and mathematics competence

There is a consensus among scholars that both home and school environments play key roles in facilitating students’ mathematics competence. For instance, a systematic review established a close correlation between home possession and students’ competence in mathematics (Wang et al., 2023). Other existing studies referred to home possession as cultural capital and explained that having access to it significantly resulted not only in higher competence but also higher expectations in mathematics-related jobs (Cascella, 2019; Holmes et al., 2018). Particularly, a substantial relationship has been established between resources at home and South African students’ mathematical competence in TIMSS 2015 (Mensah, 2020). This implies that the higher the number of learning resources students have access to at home, the higher their competency level.

The school environment variables considered in this study are the instructional environment, sense of belonging, and bullying. The role of sense of belonging is clearly elucidated in a study that suggested that a higher level of sense of belonging in school served as a potential for reducing mathematics anxiety and, hence, improving competence (Wang, 2024). The sense of belonging is imperative, as it prepares students psychologically to engage in instruction. This statement is especially true when students feel safe to take intellectual risks, like asking questions or trying to solve hard problems without worrying about being made fun of (Arends and Visser, 2019). On the contrary, in a school environment where students feel bullied, threatened, and insecure, there is a higher possibility of their not developing academic competencies, including mathematics competence. This premise is corroborated by an extant study that revealed a direct negative link between school bullying and mathematics competence (Ren et al., 2025). In their study, the researchers suggested that the effect of school bullying was more severe for female students, a situation that needs to be addressed because of the advocacy to attract women to the field of mathematics (Cavicchi, 2025; Paudel et al., 2025; Rossi et al., 2022). Bullying in all forms, whether traditional or cyber, predicted reduced mathematical competence (Graham, 2025) and had both long-term and short-term influence on learners (Camodeca and Nava, 2022). Moreover, the instructional environment can predict whether students will be mathematically competent. Extant literature identified the instructional environment as a vital mechanism for triggering instructional enjoyment, hence academic success (Liu et al., 2022). Additionally, according to Wilson Fadiji and Reddy (2023), clarity of instruction promoted students’ engagement, thereby leading to enhancement in their competence level. Students’ subjective perception of their instruction can influence the way they assimilate knowledge, which translates into their competence (Hawk et al., 2025).

In sum, the existing literature demonstrates that both home and school environments are meaningfully associated with students’ mathematics competence. However, much of this evidence has examined these contextual factors in relation to overall mathematics competence, implicitly treating students as a relatively homogeneous group. Consequently, less is known about whether the role of contextual conditions differs across students who occupy distinct levels of mathematics competence. This gap is particularly important because a contextual factor that is associated with average mathematics competence may not be equally relevant for students at different levels of competence distribution. Building on this gap, the present study first uses latent profile analysis to identify distinct mathematics-competence profiles and subsequently examines whether home and school contextual characteristics are associated with membership in these profiles.

## 3 Method

The present study includes Grade 8 students from South Africa who participated in the TIMSS 2023 assessment. TIMSS is a large-scale international comparative assessment that evaluates students’ performance in mathematics and science every 4 years (Fishbein et al., 2025). TIMSS also has rich data on students’ backgrounds, learning attitudes, and school and classroom environments. The initial sample was 11,940 students. The study excludes students with missing responses on background, mathematics attitude, and home and school environments. A total of 928 students were excluded, resulting in a final sample of 11,012 students consisting of 5,511 (50.05%) females and 5,501 (49.95%) males. The mean age of the students is 15.76 years (SD = 4.14).

### 3.1 Ethical considerations

Since the study utilizes anonymized human participant data from the IEA, the current researchers made no direct contact with the participants. All identifiable information was removed prior to public release, ensuring participants’ confidentiality and privacy. The analysis complied fully with the ethical use of secondary data, and the researchers adhered to all conditions governing the use of TIMSS data for academic and non-commercial purposes.

### 3.2 Measures

#### 3.2.1 Mathematics competence

The study assessed mathematics competence using seven TIMSS mathematics indicators: Number, Algebra, Geometry, Data and Probability, Knowing, Applying, and Reasoning. TIMSS provides five plausible values (PVs) for each mathematics indicator, which are randomly drawn from the posterior probability distribution to represent the uncertainty surrounding students’ achievement estimates (Aldrich et al., 2024). In the present study, the five plausible values were retained across the five imputed datasets, and the seven mathematics indicators were entered jointly into the latent profile analysis to identify distinct profiles of mathematics competence.

#### 3.2.2 Person-level variables

The present study includes some person-level variables as predictors of membership in mathematics competence profiles. The variables include sex, age, students’ liking learning mathematics (math enjoyment), confidence in mathematics (math confidence), and valuing mathematics (math value).

#### 3.2.3 Context-level variables

Context-level variables include home educational resources (home resources). Home resources include the number of books students have at home and whether they own certain digital devices. School-level variables include instructional environment (negative instructional environment), instructional clarity in mathematics lessons (math instructional clarity), students’ sense of school belonging (belonging), and bullying in school (bullying). Table 1 presents all predictors and their coding.

**Table 1:** Variable description.

Variable	Description	Measurement and analytical coding
Person		
Sex	Student sex	Binary; 1 = female, 0 = male
Age	Students’ age at the time of assessment	Continuous; age in years
Mathematics confidence	Students’ confidence in performing mathematical tasks	Ordinal source responses: Very Confident → Somewhat Confident → Not Confident (Reversed coded).
Mathematics value	The extent to which students value learning mathematics	Ordinal source responses: Strongly Value → Somewhat Value → Do Not Value (Reversed coded).
Mathematics enjoyment	Students’ enjoyment of learning mathematics	Ordinal source responses: Very Much Like Learning Mathematics → Somewhat Like Learning Mathematics → Do Not Like (Reversed coded)
Context		
Home educational resources	Availability of educational resources at home, including books, computers/tablets, smartphones, internet access, study desk, and related resources	Continuous TIMSS scale score reflecting the availability of home educational resources
Instructional clarity	Students’ perceptions of the clarity of mathematics instruction	Ordinal source responses reflecting High, Moderate, and Low Clarity of Instruction (Reversed coded).
Disorderly behavior	Students’ reports of disorderly behavior by other students during mathematics lessons	Ordinal source responses: Few or No Lessons → Some Lessons → Most Lessons (Reversed coded).
Sense of school belonging	Students’ sense of belonging in their school	Ordinal source responses: Little Sense of School Belonging → Some Sense of School Belonging → High Sense of School Belonging.
Bullying	Frequency with which students experienced bullying at school	Ordinal source responses: Never or Almost Never → About Monthly → About Weekly.

### 3.3 Data analysis

Prior to the main analysis, a preliminary multicollinearity assessment was conducted using variance inflation factors (VIFs) and tolerance values. Following conventional benchmarks, VIF values below 5 and tolerance values above 0.20 were considered indicative of acceptable levels of predictor overlap (Kline, 2018).

To address the first research question, a person-centered Latent Profile Analysis (LPA) was conducted to identify distinct patterns of mathematics competence among South African Grade 8 students. The analysis used seven TIMSS mathematics achievement indicators comprising the two dimensions of the TIMSS mathematics framework. The content dimension was represented by Number, Algebra, Geometry, and Data and Probability, which describe the mathematical subject matter assessed. The cognitive dimension was represented by Knowing, Applying, and Reasoning, which describe the cognitive processes students draw upon when working with mathematical content. The five plausible values available for each of the seven mathematics indicators were retained as separate imputations and analyzed simultaneously in Mplus 8.3 using the multiple-imputation framework, thereby incorporating uncertainty associated with the plausible values. The TIMSS student sampling weight (TOTWGT) was incorporated in the analyses to reflect the differential probabilities of selection and produce weighted estimates.

Models containing two to five profiles were estimated. Model enumeration was guided by the joint consideration of AIC, BIC, SABIC, entropy, average posterior classification probabilities, profile prevalence, parsimony, and substantive interpretability. Lower values of AIC, BIC, and SABIC indicate better relative fit, whereas higher entropy and posterior probabilities indicate better classification (Berlin et al., 2014; Spurk et al., 2020). Importantly, no single criterion was treated as sufficient for determining the final number of profiles. In particular, solutions were evaluated according to whether additional profiles represented meaningfully distinct achievement configurations or merely divided an existing competence continuum into increasingly finer segments. This was particularly relevant given the large sample, in which information criteria can continue to improve as additional profiles are introduced. The final solution is therefore selected by balancing statistical improvement against classification quality, parsimony, profile prevalence, and the conceptual coherence of the resulting competence structure.

To address the second research question, latent profile membership was modeled as the outcome in a multinomial logistic regression framework using the three-step R3STEP procedure. This approach examines characteristics associated with membership in the empirically derived mathematics-competence profiles while preserving the measurement of the profiles themselves. Student sex, age, home educational resources, mathematics-related attitudes, and school-related experiences are included as predictors. The first profile is specified as the reference category, and associations are reported as odds ratios (ORs) with 95% confidence intervals. The R3STEP analysis is conducted using the same five plausible-value datasets and student sampling weight. Because Mplus 8.3 does not permit replicate-weight estimation with TYPE = MIXTURE; the mixture models were estimated using the student sampling weight rather than the replicate weights.

## 4 Results

### 4.1 Multicollinearity check

Table 2 presents the results of the multicollinearity assessment. VIFs ranged from 1.00 to 4.79 (M = 3.08), while tolerance values ranged from 0.209 to 0.996, indicating no problematic multicollinearity among the predictors.

**Table 2:** Multicollinearity diagnostics for predictors.

Predictor	VIF	Tolerance (1/VIF)
Sex	1.00	0.996
Home educational resources	1.01	0.994
Like learning mathematics	4.79	0.209
Mathematics confidence	3.94	0.254
Value mathematics	2.79	0.359
Instructional clarity	4.23	0.237
Disorderly behavior	4.51	0.222
School belonging	2.01	0.497
Bullying	3.47	0.288
Mean VIF	3.08	—

VIF, Variance inflation factor. Tolerance = 1/VIF.

### 4.2 Latent profiles of mathematics competence

The LPA revealed substantial heterogeneity in South African students’ mathematics achievement. As shown in Table 3 and Figure 1, the information criteria improved progressively from 2 through 5 profiles. AIC decreased from 931,305.481 in the 2-profile solution to 890,513.637 in the 5-profile solutions. BIC declined from 931,467.888 to 890,853.213, while SABIC declined from 931,397.974 to 890,707.031.

**Table 3:** Latent profile model fit and classification indices for two- to five-profile solutions.

Fit index	2 profiles	3 profiles	4 profiles	5 profiles
AIC	931,305.481	908,325.913	896,568.423	890,513.637
BIC	931,467.888	908,547.376	896,848.943	890,853.213
SABIC	931,397.974	908,452.039	896,728.183	890,707.031
Entropy	0.914	0.914	0.907	0.896
Smallest profile (%)	32.36	13.00	7.80	4.36

AIC, Akaike information criterion; BIC, Bayesian information criterion; SABIC, sample-size adjusted Bayesian information criterion. Lower information-criterion values indicate better relative fit.

**Figure 1:**
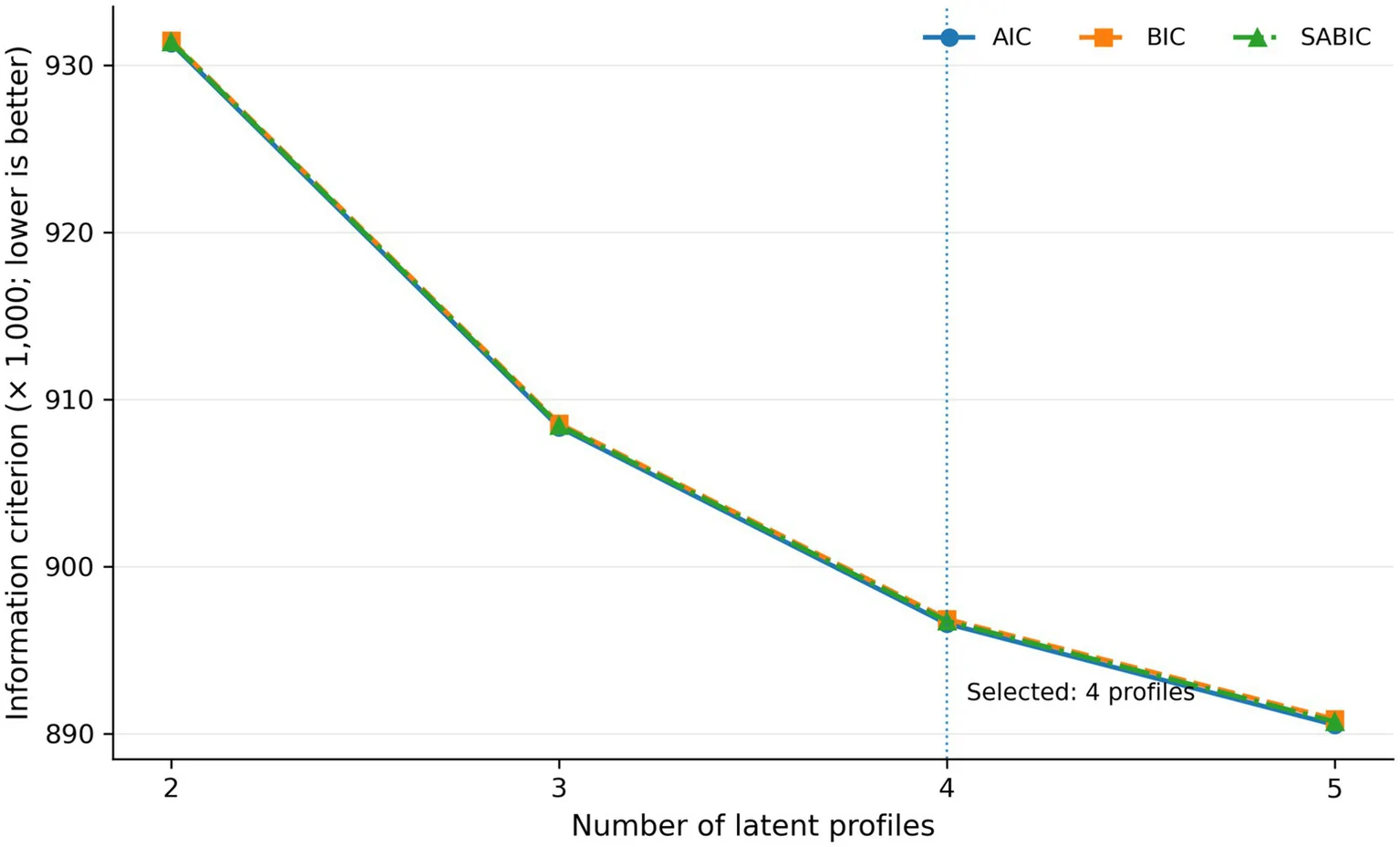
Model fit across latent profile solutions.

The 4-profile solution provided a substantial improvement over both the 2- and 3-profile solutions while retaining high classification quality. Entropy was 0.907, and average posterior probabilities for the most likely class ranged from 0.940 to 0.972 across the four profiles. The estimated profile proportions were 23.47, 25.73, 43.00, and 7.80%, with the corresponding most-likely classification proportions differing only minimally. The five-profile solution yielded further reductions in AIC, BIC, and SABIC and therefore represented a statistically competitive alternative. However, the smallest profile represented only 4.36%, while the solution’s incremental profile did not provide a sufficiently compelling substantive gain to warrant the additional complexity. Entropy declined from 0.907 to 0.896, while the fifth solution divided the achievement continuum into an additional intermediate subgroup rather than producing a qualitatively different pattern of strengths and weaknesses across the mathematics indicators. The 4-profile solution, therefore, offered the most parsimonious representation of the achievement structure while retaining strong classification and clearly interpretable competence levels.

#### 4.2.1 Profile characteristics

The retained four-profile model demonstrated strong classification quality (entropy = 0.907), with average posterior probabilities of 0.947, 0.946, 0.940, and 0.972 for the Low, Lower-middle, Upper-middle, and High profiles, respectively, indicating clear separation among the profiles. The profiles comprised 23.47% of students in the Low, 43.00% in the Lower-middle, 25.73% in the Upper-middle, and 7.80% in the High mathematics-competence profile. As illustrated in Table 4 and Figure 2, the four profiles showed a consistent ordering across all seven mathematics achievement indicators, with the Low profile displaying the lowest achievement (244.87–325.65), followed by the Lower-middle (324.49–396.20) and Upper-middle profiles (403.38–473.04), while the High profile consistently showed the highest achievement (532.79–575.45). The result revealed a monotonic pattern across Algebra, Geometry, Data and Probability, Knowing, Applying, and Reasoning. This consistent ordering across both the content domains and cognitive domains provides strong substantive coherence for the 4-profile solution, indicating that the profiles represent progressively differentiated levels of mathematics competence.

**Table 4:** Mean of mathematical competence for each profile.

Mathematics indicator	Low 23.47%	Lower-middle 43.00%	Upper-middle 25.73%	High 7.80%
Number	325.65	395.94	465.57	561.44
Algebra	318.22	396.20	473.04	575.45
Geometry	257.12	340.86	423.02	552.02
Data and probability	244.87	324.49	403.38	532.79
Knowing	310.13	379.61	457.48	565.26
Applying	318.87	382.62	452.28	554.24
Reasoning	286.22	359.46	438.38	557.74

**Figure 2:**
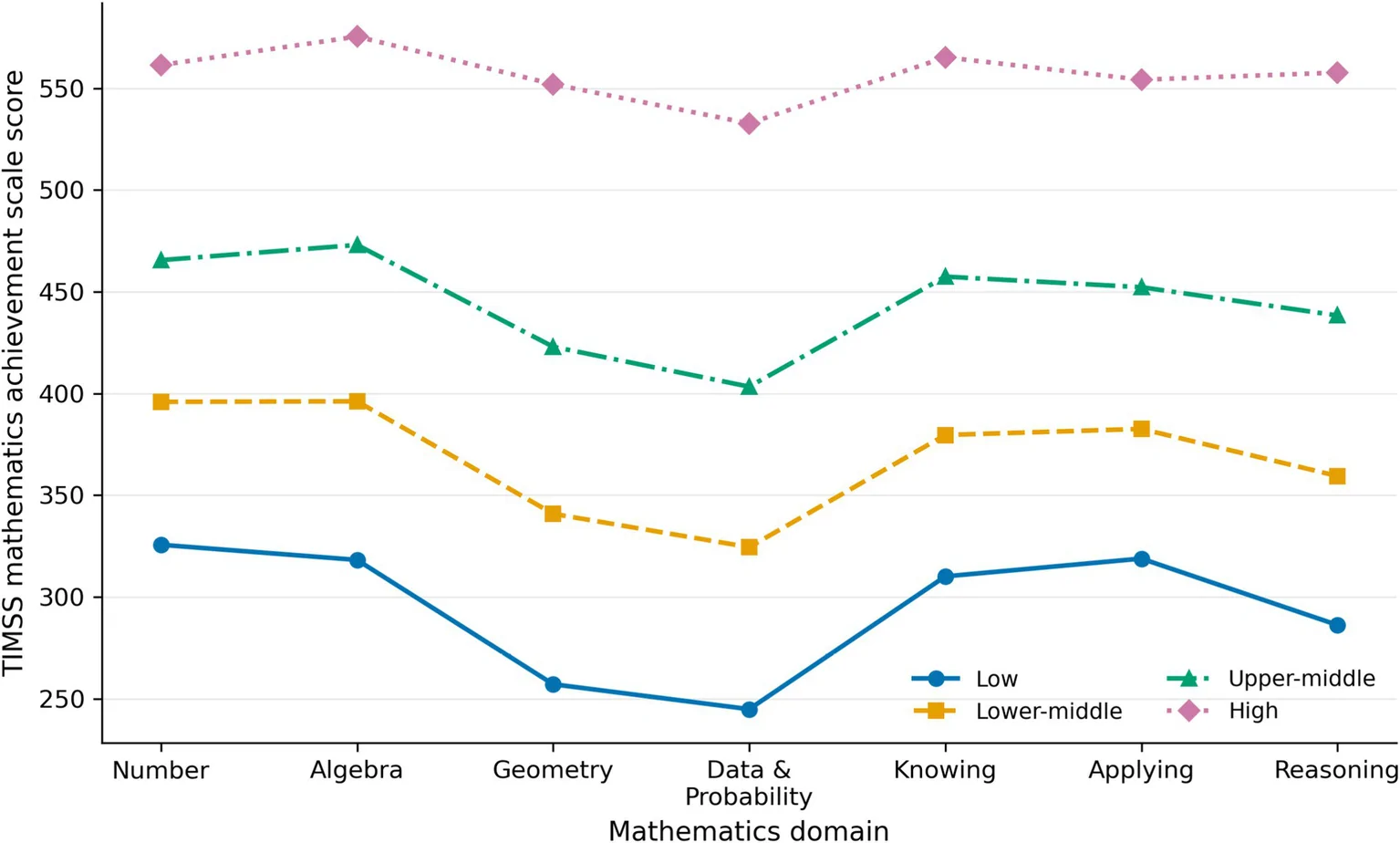
Mathematics competence profile.

### 4.3 Predictors of mathematics-competence profile membership

The R3STEP analysis indicated that ecological characteristics were differentially associated with mathematics-competence profile membership. At the person level, age was negatively associated with membership in all three higher-competence profiles relative to the Low-competence profile. Relative to the Low profile, a one-unit increase in age was associated with lower odds of membership in the Lower-middle (OR = 0.645, *p* < 0.001), Upper-middle (OR = 0.467, *p* < 0.001), and High (OR = 0.506, *p* < 0.001) profiles. The effect sizes indicated meaningful differences in profile membership: a one-unit increase in age corresponded to approximately 35.5, 53.3, and 49.4% lower odds of belonging to the Lower-middle, Upper-middle, and High profiles, respectively, relative to the Low profile. Mathematics-related attitudes showed more differentiated patterns. Mathematics confidence was positively and significantly associated with the Upper-middle (OR = 1.340, *p* < 0.001) and High (OR = 1.915, *p* < 0.001) profiles, indicating that a one-unit increase in confidence was associated with approximately 34 and 92% higher odds, respectively, of belonging to these profiles rather than the Low profile. Valuing mathematics was had positive association with Lower-middle (OR = 1.211, *p* < 0.001) and Upper-middle (OR = 1.159, *p* = 0.001) profiles. Surprisingly, a one-unit increase in reported enjoyment of mathematics was associated with 21.2% lower odds of belonging to the High-competence profile relative to the Low-competence profile (OR = 0.788, *p* < 0.001). However, enjoyment of mathematics was not significantly associated with membership in the Lower-middle or Upper-middle profiles. Meanwhile, sex was not significantly associated with membership in any of the higher-competence profiles relative to the Low-competence profile.

At the context level, the analysis revealed that home educational resources were associated with higher odds of belonging to the Upper-middle (OR = 1.242, *p* < 0.001) and High (OR = 1.984, *p* < 0.001) profiles. Thus, a one-unit increase in home educational resources was associated with almost twice the odds of belonging to the High profile rather than the Low profile. Similarly, school belonging showed significant positive association with all three higher-competence profiles: a one-unit increase in the belonging scale was associated with 51% higher odds of Lower-middle membership (OR = 1.513, *p* < 0.001), approximately twice the odds of Upper-middle membership (OR = 2.002, *p* < 0.001), and 130% higher odds of High-profile membership (OR = 2.295, *p* < 0.001). On the contrary, experiencing bullying in school was negatively associated with membership in the Lower-middle (OR = 0.883, *p* < 0.001), Upper-middle (OR = 0.690, *p* < 0.001), and High (OR = 0.689, *p* < 0.001) profiles. Finally, instructional clarity and disorderly behavior were not significantly associated with profile membership. Overall, the findings suggest that mathematics-competence profiles were associated with a combination of student characteristics and characteristics of students’ immediate learning environments, although the magnitude and direction of these associations differed across levels of competence. All results are presented in Table 5.

**Table 5:** Predictors of mathematics-competence profile membership.

Predictor	Lower-middle vs. Low OR (95% CI)	Upper-middle vs. Low OR (95% CI)	High vs. Low OR (95% CI)
Person			
Sex	1.001 (0.852, 1.176)	0.910 (0.761, 1.088)	1.203 (0.883, 1.640)
Age	0.645*** (0.605, 0.688)	0.467*** (0.445, 0.490)	0.506*** (0.464, 0.552)
Like learning mathematics	0.991 (0.895, 1.097)	1.022 (0.936, 1.116)	0.788*** (0.710, 0.874)
Mathematics confidence	1.070 (0.984, 1.164)	1.340*** (1.189, 1.510)	1.915*** (1.514, 2.423)
Value mathematics	1.211*** (1.089, 1.346)	1.159** (1.055, 1.273)	1.009 (0.901, 1.130)
Context			
Home educational resources	1.053 (0.995, 1.115)	1.242*** (1.151, 1.341)	1.984*** (1.565, 2.515)
Instructional clarity	1.031 (0.965, 1.102)	1.044 (0.981, 1.112)	0.974 (0.888, 1.068)
Disorderly behavior	1.005 (0.948, 1.066)	1.002 (0.923, 1.088)	1.083 (0.984, 1.192)
School belonging	1.513*** (1.293, 1.770)	2.002*** (1.595, 2.513)	2.295*** (1.700, 3.098)
Bullying	0.883*** (0.837, 0.931)	0.690*** (0.660, 0.722)	0.689*** (0.642, 0.739)

OR, odds ratio; CI, confidence interval. The Low-competence profile was the reference category. ORs reflect the change in odds associated with a one-unit increase in the predictor. *p < 0.05; **p < 0.01; ***p < 0.001.

## 5 Discussion

Several studies have examined how personal and contextual factors are associated with South African students’ mathematics competence, particularly using large-scale assessments such as TIMSS. However, much of this research treated students as a relatively homogeneous group, potentially obscuring meaningful differences in how they are distributed across levels of mathematics competence. The present study extends the literature by identifying distinct patterns of mathematics competence through LPA and subsequently examining factors associated with students’ membership in these profiles through the lens of Bronfenbrenner’s ecological theory of human development. The results revealed four profiles of participating students, namely, Low, Lower-middle, Upper-middle, and High mathematics competence, and showed that profile membership was differentially associated with person-level characteristics and features of students’ immediate learning environments.

### 5.1 Profiles of mathematics competence

The person-centered logic underlying LPA is that aggregate, sample-wide estimates of mathematics competence can obscure more than they reveal (Howard and Hoffman, 2018; Spurk et al., 2020). Where a variable-centered regression asks how competence, on average, relates to a set of predictors, a person-centered analysis asks what configurations of competence actually exist among students, treating the individual rather than the variable as the unit of interest, which is a holistic-interactionist framing in which the person is treated as a system to be understood as a whole rather than decomposed into isolated variables. The 4-profile solution retained in this study suggests that South African Grade 8 students’ mathematics competence is not usefully summarized by a single national mean but is instead organized into ordered strata that differ from one another by magnitude rather than by kind. The profiles moved in lockstep across all seven content and cognitive indicators rather than trading strengths for weaknesses across domains. This finding corroborates findings by previous research, which demonstrates different profiles of students based on their mathematical competence (Zeng, 2024).

The distribution of students across those profiles is the more consequential finding. Fewer than one in 10 students (7.80%) fell into the High profile, while close to two-thirds were split between the Low and Lower-middle profiles. This pattern sits comfortably within a well-established line of South African education research documenting a persistently skewed, two-tier achievement structure in which a small, well-resourced minority performs at internationally competitive levels while the majority cluster far below grade-appropriate benchmarks (Muyambi and Ahiaku, 2025; Spaull and Kotze, 2015). Although 7.80% of the sample scored above 500 points across all dimensions, the majority of them scored below 400 points. It is therefore not surprising that the country’s average performance was ranked in the last five positions (Von Davier et al., 2024). Yet a national ranking of this kind, read on its own, risks being taken as evidence of uniformly weak learning across the board. The present analysis complicates that reading. The High profile identified here was small, 7.80% of the sample, though numbering well over 800 students, but its members achieved at levels (532.79–575.45 across indicators) that would be internationally competitive by any standard. The low national mean, then, is not a description of a uniformly struggling population; it is, in part, an artifact of pooling a large low-performing majority with a much smaller but genuinely high-performing minority, a distinction that a single aggregate score cannot preserve.

### 5.2 Predictors of profile membership

The finding that age correlated negatively with higher profile membership is consistent with Bansilal and Lephoto (2022) finding, which suggested that among some South African students, older ones were less likely to score higher in mathematics. This finding, however, contradicts evidence from other contexts that age relates positively with reasoning capacity and, by extension, competence (Almomani et al., 2018; Akin, 2025). The likely reconciliation is that age is not doing the same conceptual work in all contexts. In systems with limited grade repetition, age variation largely reflects developmental maturation, especially if repetition and delayed enrollment are common; age-for-grade may be a marker of prior schooling disruption rather than cognitive development. Meanwhile, sex notably distinguished none of the profiles, which is worth pausing on given how much attention sex has received in the literature, including findings of a pro-girl gap in South African TIMSS performance (Hofmeyr, 2022) and profile-specific gender effects on reasoning items (Sibanda and Naidoo, 2023). These results suggest that demographic characteristics may operate differently across ecological contexts, with age appearing to capture aspects of students’ educational histories in addition to developmental maturity. From an ecological perspective, the absence of sex differences across profiles further suggests that mathematics competence may be shaped less by a single person-level characteristic and more by the interplay between individual characteristics and students’ immediate learning environments.

Meanwhile, confidence and the value students place on mathematics, by contrast, distinguished the higher-competence profiles in the direction the broader literature would predict, reinforcing a relationship between affective orientation and competence that has proven durable across settings (Flanagan and Einarson, 2017; Nakamoto et al., 2025). What did not fit expectations was enjoyment, which was associated with lower rather than higher odds of belonging to the High profile. These findings partially echoes claims by Raabe and Block (2024), which indicated that confidence and achievement do not always move together in the same direction for all learners, specifically for boys, whose overestimated confidence carried an adverse rather than a protective effect. From an ecological perspective, this pattern suggests that mathematics-related attitudes are not uniformly associated with competence but may operate differently across levels of the competence continuum and within students’ immediate learning environments. The stronger associations for confidence and value at the upper end of the profiles indicate that students’ individual orientations toward mathematics may become increasingly salient in distinguishing higher levels of competence, whereas the unexpected pattern for enjoyment points to a more complex relationship that may depend on students’ broader educational experiences and contexts.

Where person-level characteristics distinguished profiles unevenly, the immediate learning environment was more consistent. Home educational resources separated the higher-competence profiles from the Low profile in a manner that is in line with the cultural- and material-capital literature, which has long treated resource access at home as a proxy for the kind of sustained, cumulative advantage that compounds over a schooling period (Wang et al., 2023; Cascella, 2019; Holmes et al., 2018) and which Mensah (2020) documented specifically for South African TIMSS 2015 mathematics outcomes. School belonging showed a comparable but arguably more interesting pattern by separating each profile from the one beneath it in a graded manner, consistent with belonging functioning less as a threshold condition and more as an accumulating psychological resource, the kind that prepares students to take the intellectual risks (i.e., attempting a hard problem or asking a clarifying question) that Arends and Visser (2019) identify as a prerequisite to deeper engagement and that Wang (2024) links to reduced mathematics anxiety. Bullying moved in the expected opposite direction, weakening the odds of higher-profile membership in a way that corroborates a substantial body of literature linking peer victimization to reduced mathematics outcomes (Ren et al., 2025; Graham, 2025; Camodeca and Nava, 2022). The clearer unexpected finding was the non-significant result for instructional clarity, a variable that features prominently in the literature as a driver of engagement and, through it, competence (Wilson Fadiji and Reddy, 2023). Perhaps instructional clarity may be more relevant to how students experience and engage with instruction than to distinguishing students at different levels of mathematics competence. The lack of a significant association therefore suggests that clear instruction alone may not differentiate competence profiles once person-level characteristics and other contextual factors are considered.

### 5.3 Implications

The findings highlight the importance of recognizing heterogeneity in students’ mathematics competence when designing educational support. The four profiles highlighted meaningful differences in the factors associated with membership. Notably, the sense of belonging in school showed the strongest and most consistent associations with profile membership, with higher belonging scores corresponding to progressively greater odds of belonging to the higher-competence profiles. The directions of sense of belonging and bullying in relation to profile membership reinforce the importance of cultivating welcoming and inclusive school environments in which students feel respected, supported, and safe to engage with both peers and teachers (Arends and Visser, 2019; Wang, 2024). However, given the unexpected direction of the association between mathematics enjoyment and profile membership, the finding should not be interpreted as evidence that enjoying mathematics reduces mathematics competence; rather, it suggests that the relationship between mathematics enjoyment and competence may be more complex and context-dependent than a simple positive association would imply.

In practice, teachers and school leaders could use students’ differing competence patterns to inform differentiated support, including strengthening mathematics confidence and value, improving access to educational resources, and fostering inclusive school environments in which students feel that they belong and can seek academic and social support. Such strategies should be viewed as context-sensitive and potentially beneficial approaches. Schools need to identify students who may require additional academic or psychosocial support while avoiding one-size-fits-all approaches to mathematics learning. Ultimately, efforts should focus on creating learning environments that respond to students’ differing needs and provide opportunities for both struggling and higher-achieving students to progress.

Theoretically, the findings extend the application of the ecological theory to mathematics competence by showing that differences in students’ achievement are associated with both person-level characteristics and features of their immediate learning environments (Bronfenbrenner, 1979; Uddin, 2017), rather than being reducible to individual ability alone. The identification of four progressively differentiated competence profiles further suggests that the relationship between individual characteristics and mathematics achievement is not uniform across students. In particular, the stronger role of mathematics confidence, the value attached to mathematics, home educational resources, and school belonging among higher-competence profiles highlights the importance of considering students within their broader developmental contexts. The findings therefore reinforce the ecological proposition that learning outcomes emerge through the interaction between students and the environments in which their learning takes place (Tudge et al., 2009), while also suggesting that these associations may vary across levels of competence rather than operating uniformly across the student population.

### 5.4 Limitations and suggestion for future research

The current study has some limitations that should be considered when interpreting the findings. First, although the study drew on a large-scale assessment designed to provide population-level evidence, the analysis was based on sampled South African students and therefore should be interpreted within the population and sampling scope of the assessment rather than assumed to represent every student. Second, latent profile membership is probabilistic, and although the selected four-profile solution demonstrated strong classification quality, some degree of classification uncertainty remains inherent in LPA. Third, several predictors, particularly students’ mathematics-related attitudes and experiences of the school environment, were based on self-reported information and may, therefore, be affected by recall, social-desirability, or perception-related bias. Future research could complement student reports with teacher reports, administrative information, or direct observations. Fourth, the cross-sectional design limits conclusions about temporal ordering and causality; the observed associations should therefore not be interpreted as evidence that the identified factors cause differences in mathematics competence. Longitudinal studies would be better positioned to examine developmental changes and potential causal pathways. Finally, although the study incorporated theoretically relevant person- and contextual characteristics, other potentially important influences on mathematics competence were not measured. Future research could incorporate additional family, instructional, school, and socioeconomic factors to provide a more comprehensive explanation of differences in mathematics-competence profile membership.

## 6 Conclusion

The overall performance of South African students in TIMSS 2023 mathematics was unsatisfactory, prompting scholars to investigate the factors influencing their performance and explore ways to address the academic challenges these students face. Meanwhile, many scholars treat students as a homogeneous group. The current study, however, started with the assumption that students at different levels of mathematics competence may require varying forms of support to address their mathematical challenges. Guided by Bronfenbrenner’s ecological theory of human development, the study examined person-level characteristics and features of students’ immediate home and school environments using LPA and multinomial logistic regression. The results indicated that the students included in the study could be categorized into four profiles based on their mathematics competence, namely Low, Lower-middle, Upper-middle, and High. In addition, the study revealed varying magnitudes and directions of associations between the predictors and profile membership, with some characteristics differentiating particular profiles but not others. The study highlights the importance of taking into account the heterogeneity of students when planning educational policies and interventions so that support can be differentiated according to students’ varying levels of mathematics competence and some groups are not overlooked.

## Data Availability

Publicly available datasets were analyzed in this study. This data can be found at: https://www.iea.nl/studies/iea/timss/timss2023.
